# Supplementary material for: How group structure impacts the numbers at risk for coronary artery disease: polygenic risk scores and nongenetic risk factors in the UK Biobank cohort
Source: Genetics. 2024 May 23;227(3):iyae086. doi: 10.1093/genetics/iyae086 (PMC11339605; doi:10.1093/genetics/iyae086)
Supplement: iyae086_Supplementary_Data [file iyae086_supplementary_data.pdf]

Table S1: Definition of Coronary Artery Disease (CAD) following Elliott et al. (2020).

ICD-10	ICD-9	OPCS-4	Non-cancer illness code (Biobank field: 20002)	Operation code (Biobank field: 20004)	Vascular/heart problems (Biobank field: 6150)
I21	410	K40.1-4	1075: Heart attack/myocardial infarction	1070: Coronary angioplasty	1: Heart attack
I22	411	K41.1-4		1095: Coronary artery bypass grafts	
I23	412	K45.1-5			
I24.1	427.89*	K49.1-2			
I25.2		K49.8-9			
		K50-2			
		K75.1-4			
		K75.8-9			

* Changed 429.79 from Elliot et al. (2020) eTable 2 to 427.89, as we believed this was a typo in their table.

Table S2: UKB hospital inpatient record data fields and corresponding date fields; if a participant has more than one listed event, retain only the first CAD.

Data type	Data category	UKB fields	Corresponding date fields	No. prevalence CAD	No. incidence CAD	Lacking date information
Hospital inpatient data	ICD-10	41270	41280	6692	18013	0
	ICD-9	41271	41281	195	0	0
	OPCS-4	41272	41282	8081	14273	0
self-reported	Non-cancer illness code	20002	20009	11139	990	0
	Operation code	20004	20010	7783	1311	0
	Vascular/heart problems	6150	3894 :Age heart attack diagnosed	11140	642	64

Table S3: Number of first-ever CAD prevalence and incidence events from inpatient and self-reported records.

	No. prevalence CAD*	No. incidence CAD	Can't define**	Total
Inpatient record	11339	21469	0	32808
Self-reported record	14840	1585	13	16438
Combined	16546	19047***	12	35605

* Prevalence CAD events occurred before the date participants visited the baseline assessment centres.

** Due to lack of information on CAD onset date/age

*** The combined number of incidence CAD events is lower than the number from the hospital records because many participants reported CAD events in their self-reports, but these events were not recorded in the hospital records.

Table S4: ASCVD UKB fields for selection of prevalent ASCVD events. The recorded event time (or age) was compared with the date (or age) when each participant joined the UKB, any events occurring before this date were counted as prevalent ASCVD events.

Elliott et al. 2020 definition of cardiovascular disease (CVD)					
ICD-10	ICD-9	OPCS-4	Non-cancer illness code	Operation code	Vascular/heart problems
G45	410	K40	1075: Heart attack/myocardial infarction	1070: Coronary angioplasty	1
I20	411	K41	1082: Transient ischaemic attack	1071: Other arterial surgery/ revascularisation procedures	2
I21	412	K42	1583: Ischaemic stroke	1105: Carotid artery surgery/endarterectomy	3
I22	413	K43		1109: Carotid artery angioplasty +/- stent	
I23	414	K44		1514: Coronary angiogram	
I24	434	K45			
I25	436	K46			
I63		K47.1			
I64		K49			
		K50			
		K75			
Klarin et al. 2019 definition of peripheral artery disease (PAD)					
I70.0	4400	X09.3	1067	1102	
I70.00	4402	X09.4	1087	1108	
I70.01	4438	X09.5	1088	1440	
I70.2	4439	L21.6			
I70.20		L51.3			
I70.21		L51.6			
I70.8		L51.8			
I70.80		L52.1			
I70.9		L52.2			
I70.90		L54.1			
I73.8		L54.4			
I73.9		L54.8			
		L59.1			
		L59.2			
		L59.3			
		L59.4			
		L59.5			
		L59.6			
		L59.7			
		L59.8			
		L60.1			
		L60.2			
		L63.1			
		L63.5			
		L63.9			
		L66.7			

Table S5: Variables included in the PCE scores calculation.

Variables	UKB field used	Additional explanation
Age when attended assessment centre	21003	
Initial assessment centre visit	54	
Total cholesterol	30690	Took the results from Initial assessment visit (2006-2010), change units of measurement from mmol/L to mg/dL, 1 mmol/L = 18 mg/dL
HDL cholesterol	30760	Took the results from Initial assessment visit (2006-2010) , change units of measurement from mmol/L to mg/dL, 1 mmol/L = 18 mg/dL
Systolic blood pressure	4080	Took the results from Initial assessment visit (2006-2010)<br>If two values were taken at their initial visit, we used the average value; otherwise used the only value there.
Smoking status	20116	
Hypertension medication	Elliotts et al. 2020, eTable 1,<br>Blood pressure lowering medications<br><br>6177 & 6153 : 2,<br><br>20003:<br>1140860192, 1140860292, 1140860696, 1140860728, 1140860750, 1140860806, 1140860882, 1140860904, 1140861088, 1140861190, 1140861276, 1140866072, 1140866078, 1140866090, 1140866102, 1140866108, 1140866122, 1140866138, 1140866156, 1140866162, 1140866724, 1140866738, 1140868618, 1140872568, 1140874706, 1140874744, 1140875808, 1140879758, 1140879760, 1140879762, 1140879802, 1140879806, 1140879810, 1140879818, 1140879822, 1140879826, 1140879830, 1140879834, 1140879842, 1140879866, 1140884298, 1140888552, 1140888556, 1140888560, 1140888646, 1140909706, 1140910442, 1140910614, 1140916356, 1140923272, 1140923336, 1140923404, 1140923712, 1140926778, 1140928226, 1141145660, 1141146126, 1141152998, 1141153026, 1141164276, 1141165470, 1141166006, 1141169516, 1141171336, 1141180592, 1141180772, 1141180778, 1141184722,	

	1141193282, 1141194794, 1141194810	
Diabetes I	20002 - 1222	
Diabetes II	20002: 1220, 1223<br><br>20003:<br>1140868902, 1140874646, 1140874674, 1140874718, 1140874744, 1140883066, 1140884600, 1141152590, 1141157284, 1141168660, 1141171646, 1141173882, 1141189090<br><br>Biobank field 2443 = 1	
Statins therapy	Followed Carter (2022)<br>20003:<br>1141146234,1140888594,1140888648,1141192410,1140861958	
